# Heat Stress in Legume Seed Setting: Effects, Causes, and Future Prospects

**DOI:** 10.3389/fpls.2019.00938

**Published:** 2019-07-31

**Authors:** Yonghua Liu, Jiajia Li, Yulei Zhu, Ashley Jones, Ray J. Rose, Youhong Song

**Affiliations:** ^1^College of Horticulture, Hainan University, Haikou, China; ^2^School of Agronomy, Anhui Agricultural University, Hefei, China; ^3^National Engineering Laboratory of Crop Stress Resistance Breeding, Anhui Agricultural University, Hefei, China; ^4^Research School of Biology, The Australian National University, Canberra, ACT, Australia; ^5^School of Environmental and Life Sciences, The University of Newcastle, Newcastle, NSW, Australia

**Keywords:** legumes, *Medicago truncatula*, heat stress, reproductive development, sugar metabolism, seed set

## Abstract

Grain legumes provide a rich resource of plant nutrition to human diets and are vital for food security and sustainable cropping. Heat stress during flowering has a detrimental effect on legume seed yield, mainly due to irreversible loss of seed number. To start with, we provide an overview of the developmental and physiological basis of controlling seed setting in response to heat stress. It is shown that every single process of seed setting including male and female gametophyte development, fertilization, and early seed/fruit development is sensitive to heat stress, in particular male reproductive development in legume crops is especially susceptible. A series of physiochemical processes including heat shock proteins, antioxidants, metabolites, and hormones centered with sugar starvation are proposed to play a key role in regulating legume seed setting in response to heat stress. The exploration of the molecular mechanisms underlying reproductive heat tolerance is in its infancy. *Medicago truncatula*, with a small diploid genome, and well-established transformation system and molecular platforms, has become a valuable model for testing gene function that can be applied to advance the physiological and molecular understanding of legume reproductive heat tolerance.

## Introduction

Legumes rank the third largest family of flowering plants (Gepts et al., 2005) and the second most important crop family, with nearly 20,000 species (Doyle and Luckow, 2003). Grain legumes provide a significant source of starch, protein, and oil, as well as flavonoids for human diets (Graham and Vance, 2003; Suso et al., 2015; Ozga et al., 2017; Song et al., 2017b). For example, soybean seed contains up to 40% protein and 20% oil, while chickpea seed contains up to 40% starch and 20% protein (Suso et al., 2015; Song et al., 2017a). Legumes have a unique capacity to fix atmosphere N due to the symbiosis between root nodules and rhizobia. Thus, legume crops are often used in rotation cultivation for sustainable agricultural systems (Zander et al., 2016; Stagnari et al., 2017). Grain legumes may be cultivated either in cool seasons such as chickpea (Siddique et al., 2013) or in the warm seasons such as soybean (Vadez et al., 2012). Notably, legume cropping is expanding to warmer regions, e.g., in India (Gaur et al., 2015). It is convenient for legume integration into annual cropping systems (Pelzer et al., 2017; Stagnari et al., 2017). Therefore, grain legumes in cropping systems play a vital role in ensuring food security for increased human population (Foyer et al., 2016; Considine et al., 2017). However, according to the IPCC report in 2018, global average temperature over the last 5 years (2014–2018) has increased by 1.04°C compared to the preindustrial base line and will reach 1.5°C as soon as by 2030 (IPCC, 2018). As a consequence, legume growth and development will be subjected to more frequent and severe heat stress as the global climate changes (Zinn et al., 2010; Vadez et al., 2012).

Heat stress during legume reproduction causes significant loss of seed yield, primarily by compromising seed setting and/or subsequent seed filling (Ruan et al., 2010; Zinn et al., 2010; Hatfield and Prueger, 2015; Siebers et al., 2015; Patriyawaty et al., 2018). It should be noted that failure of seed setting by heat stress imposed at the early reproductive stage cannot be rescued, usually leading to fatal and irreversible yield loss, while compromised seed filling by heat stress imposed at the late reproductive stage may be to some extent recovered by subsequent proper cultivation management. Heat stress during the critical period, i.e., bracketing flowering, disrupts sexual reproductive processes including microsporogenesis and megasporogenesis, pollen production and viability, stigma receptivity, pollen germination and tube elongation, fertilization and early embryogenesis (Warrag and Hall, 1984b; Monterroso and Wien, 1990; Devasirvatham et al., 2012a,b; Kumar et al., 2013; Siebers et al., 2015), inducing huge loss of seed number. For example, preanthesis heat stress impairs anther development and reduces pollen production and fertility in chickpea, while heat stress during anthesis and/or postanthesis diminishes stigma receptivity, pollen germination, and pollen tube growth, limiting the success of double fertilization (Devasirvatham et al., 2012a,b). In extreme cases, high temperature stress even causes the abscission of flower buds and flowers in common bean (Nakano et al., 1997, 1998). Heat stress just prior to or during flowering usually has the most detrimental effect on seed setting in legumes (Warrag and Hall, 1983; Prasad et al., 2008; Petkova et al., 2009; Patriyawaty et al., 2018), whereas the post-fertilization stage and early pod development may have a relatively higher tolerance to heat stress (Gross and Kigel, 1994).

Thus far, current research has predominantly focused upon morphophysiological and anatomic characteristics of floral development and fertilization under heat stress as described above. However, the physiological and molecular mechanisms of regulating legume seed setting under heat stress has received less attention, though the exploration on the molecular basis of reproductive resilience to heat stress has emerged in recent years (Paupière et al., 2014, 2017; Nahar et al., 2015; Zhang et al., 2015; Ozga et al., 2017). Therefore, this review starts to examine the heat responses of reproductive tissue development, and fertilization associated with determination of seed setting in diverse legume species, and then analyses likely physiological and molecular mechanisms by which legume seed setting withstanding heat stress have been achieved.

## Seed Setting in Response to Heat Stress

### Male Reproductive Development

Heat stress may influence every single process of anther and pollen development (Table 1). Structural abnormalities in anthers may occur under heat stress, such as changes in anther locule number and anther epidermis wall thickening in chickpea (Devasirvatham et al., 2012a, 2013). Heat stress induces a thicker exine wall and a disintegrated tapetum layer in soybean, ultimately causing abnormalities of pollen development (Djanaguiraman et al., 2012, 2013). Heat stress may lead to anther indehiscence, which prevents pollen grains shedding for pollination in cowpea and common bean (Ahmed et al., 1992; Gross and Kigel, 1994; Porch and Jahn, 2001).

**Table 1:** Effects of heat stress on the development of various male and female tissues in different legume species.

Legumes	Heat stress	Impacts on seed setting	References
Chickpea	34/19 ^o^C^a^	Anther locule number; anther epidermis wall thickening; ovule and ovary abnormality	Devasirvatham et al., 2012a, 2013
	40/25°C 32/20°C	Stigma receptivity; pollen germination; pollen tube elongation	Kumar et al., 2013; Kaushal et al., 2013
Pea	27,30,33,36°C	Pollen germination, pollen tube length, pod length, seed number per pod	Jiang et al., 2015
Cowpea	33°C	Embryo abortion; anther indehiscence	Warrag and Hall, 1984a; Ahmed et al., 1992
Soybean	38/28°C	Thicker exine wall and disintegrated tapetum layer	Djanaguiraman et al., 2012, 2013
Common bean	32/27°	Lower pollen viability; impaired female performance Pollen germination rate; anther indehiscence	Konsens et al., 1991; Ormrod et al., 1967; Gross and Kigel, 1994
Groundnut	33-48°C	Pollen sterility; retarded pollen tube growth	Prasad et al., 2001
Lupin	33/28°C	Ovule abortion	Downes and Gladstones, 1984
Lentil	35/20°C >32/20°C	Pod abortion, reduced flower number, shortened flowering period Reduced pollen viability, pollen germination, stigmatic function, ovular viability, pollen tube elongation	Bhandari et al., 2016 Sita et al., 2017a

a *Day temperature/Night temperature*.

The effects of heat stress on pollen development vary depending on the developmental time point the stress occurs. In chickpea, heat stress in early flowering leads to pollen abnormalities such as small, shrunken, and empty pollen grains (Devasirvatham et al., 2012b). Heat stress during flowering decreases pollen viability and pollen production in chickpea (Devasirvatham et al., 2012b), common bean (Konsens et al., 1991), cowpea (Ahmed et al., 1992), groundnut (Prasad et al., 1999; Kakani et al., 2002), soybean (Koti et al., 2005), and lentil (Sita et al., 2017a). Heat stress shortly after flowering disrupts the germination and tube elongation of pollens in chickpea (Devasirvatham et al., 2012b), common bean (Gross and Kigel, 1994), and soybean (Salem et al., 2007).

### Female Reproductive Development

Compared to male reproductive organs, much less is known about the effects of heat stress on female reproductive organs. The developmental failure of female organs caused by heat stress also inevitably leads to reduced legume seed set (Table 1). In common bean, heat stress during flowering causes more abnormal embryo sacs and endosperm development failure (Ormrod et al., 1967; Gross and Kigel, 1994). In lupin, heat stress causes ovule abortion and thus reduced seed set (Downes and Gladstones, 1984). In cowpea, heat stress after flowering leads to embryo abortion (Warrag and Hall, 1984a,b). In chickpea, heat stress causes ovule and ovary abnormality and thus decreased seed and pod set (Devasirvatham et al., 2013). In addition, heat stress also affects stigma receptivity and thus fertilization in chickpea (Kaushal et al., 2013; Kumar et al., 2013) and in lentil (Sita et al., 2017a).

### Different Heat Sensitivities of Male and Female Development

Similar to findings in non-legume crops such as maize, tomato, and oil rapeseed (Dupuis and Dumas, 1990; Peet et al., 1998; Young et al., 2004), it has been well documented that male reproductive development in legume crops is more susceptible to heat stress as compared to female reproductive organs, and male development is one of the major limiting factors for pod and seed set under heat stress (Kakani et al., 2002, 2005; Parish et al., 2012; Jiang et al., 2015). For example, heat stress impairs pollen development without disrupting ovule development, thus decreasing the ratio of seed/ovule in pea (Jiang et al., 2015). In chickpea, the effect of heat stress on pollen fertility starts at the third day after heat stress, whereas the effects on ovule and ovary become apparent after 7 days after heat stress (Devasirvatham et al., 2013). It has been shown that chickpea pollen grains are more sensitive to high temperature than the stigma in both field and controlled environments (Devasirvatham et al., 2013). Studies on cowpea and common bean are also in agreement with this conclusion (Warrag and Hall, 1983; Monterroso and Wien, 1990).

What is the basis of the particular sensitivity of pollen development to heat stress? Studies have shown low levels of heat shock proteins (HSPs) transcript accumulation in mature pollen of maize and its absence in pollen of *Brassica napus* (Zinn et al., 2010). Little or no increase in HSP101 protein or mRNA was observed in response to heat stress in mature maize pollen (Young et al., 2001). If there is over-expression of AtHSP101 in tobacco or cotton pollen germination and pollen tube growth under high temperature is improved (Burke and Chen, 2015). Pollen has relatively high levels of reactive oxygen species and low activity of antioxidant enzymes under heat stress in sorghum (Djanaguiraman et al., 2018). In lentil, heat tolerant genotypes were particularly associated with higher sucrose levels in pollen (Sita et al., 2017a). The interaction of HSPs reactive oxygen species (ROS), and sugar levels are discussed further below when considering physiological and molecular mechanisms of heat tolerance.

Considering the extreme sensitivity of pollen development and its importance in plant reproductive development, pollen-based screening methods have been developed to evaluate the tolerance of various genotypes to heat stress (Salem et al., 2007; Kumar et al., 2016). Based on this, many heat-tolerant lines have been developed for chickpea, common bean, faba bean, lentil, and soybean (Salem et al., 2007; Gaur et al., 2015).

### The Process of Fertilization and Early Embryogenesis

Double fertilization is a vital process by which the embryo is formed. After pollen grains have landed on the stigma, preparations for fertilization begins, with pollen germination, followed by pollen tube elongation and fusion of one sperm to the egg cell and the other sperm to the two polar nuclei of the central cell within the embryo sac, i.e., double fertilization (Dumas and Rogowsky, 2008). Heat stress could exert adverse effects on all steps of the fertilization process. Pollen germination is one of the most sensitive processes during fertilization (Saini et al., 1983). The germination rate of pollen grains was reduced by heat stress by 52% (32/27°C), 44% (43/43°C) and 50% (38/30°C) in common bean, groundnut and soybean, respectively (Porch and Jahn, 2001; Kakani et al., 2002; Koti et al., 2005). In chickpea, heat-stressed pollen grains cannot even germinate on the stigma (Devasirvatham et al., 2013). In addition to germination, the elongation of the pollen tube is also very sensitive to heat stress. In chickpea and common bean, for example, heat stress decreases pollen tube elongation, impairing the fertilization process (Ormrod et al., 1967; Gross and Kigel, 1994; Suzuki et al., 2001; Devasirvatham et al., 2012a,b; Kumar et al., 2013). In groundnut, heat stress results in not only inhibited pollen elongation, but also abnormal pollen tube growth with thinner and zigzag tubes (Prasad et al., 2001). In another study on chickpea, heat stress not only causes zigzag growth of pollen tube, but also induces pseudo-germination of pollen grains, consequently reducing fertilization (Devasirvatham et al., 2013). *In vitro* pollen germination and pollen tube elongation in soybean are significantly impaired by temperature elevated from 30/22 to 38/30°C, and heat-tolerant genotypes have greater pollen germination and tube elongation compared to heat-sensitive genotypes (Salem et al., 2007). Also, heat stress is reported to decrease pollen germination and pollen tube elongation in pea (Petkova et al., 2009; Jiang et al., 2015). Furthermore, heat-tolerant pea cultivars show a more stable composition of lipids in the pollen coat and/or exine compared to heat-sensitive cultivars (Jiang et al., 2015), which benefits the pollen-stigma interaction during the pollen germination and elongation.

However, to the best of our knowledge, most studies in legumes have focused on pollen germination and elongation, and little is known about how heat stress affects the double fertilization process and subsequent early embryogenesis. Thus, it will be necessary to draw attention to such activities, by which seed setting is significantly determined under heat stress due to its sensitivity.

## Physiological and Molecular Mechanisms of Controlling Seed Setting Under Heat Stress

**Figure 1:**
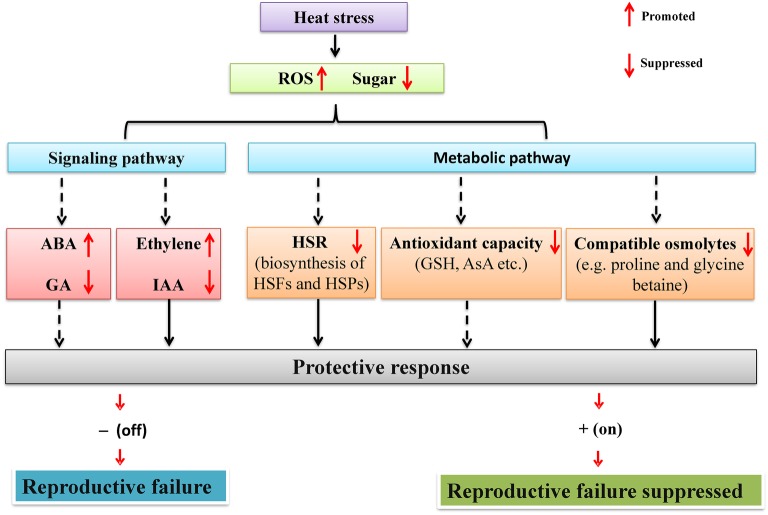
A schematic network illustrating the mechanisms by which heat stress regulates reproductive development of legumes through sugar metabolism and signaling. Under heat stress, reproductive organs of legumes experience sugar starvation. On one hand, heat stress response (HSR) (HSF and HSP) and the biosynthesis of antioxidants (GSH and AsA) and compatible osmolytes (e.g., proline and glycine betaine) are inhibited due to reduced supply of energy and carbon skeletons. On the other hand, sugar starvation also affects hormone signaling *via* sugar signaling pathways and subsequently regulates reproductive development. Solid arrows represent processes demonstrated experimentally in legumes, whereas dashed arrows indicate processes based on studies on non-legume crops. See text for more details. HSR, heat stress response; HSP, heat shock protein; HSF, heat stress transcription factor; GSH, glutathione; AsA, ascorbic acid.

Up to now, the efforts in exploring physiological and molecular basis have been mainly focused on the vegetative stage rather than reproductive stage in legumes (Lin et al., 1984; Valliyodon and Nguyen, 2006; Kumar et al., 2012; Sita et al., 2017b). However, in recent years, physiological and molecular studies of legume reproductive heat tolerance have received increased attention (Devasirvatham et al., 2012b; Kaushal et al., 2013; Sita et al., 2017b; Patriyawaty et al., 2018). For instance, in chickpea, the reproductive heat tolerance traits, i.e., eight QTLs (Table 2) for pod setting and filling have been identified, located between two markers in both CaLG05 and CaLG06. In soybean, three loci (Table 2) of flowering time under heat stress have been found in three different chromosomes (Bai et al., 2015). However, the physiological and molecular (in particular) mechanisms underlying reproductive tolerance to heat stress in legume crops are lacking. A possible reason for this is due to the lack of transgenic evidence since many legume cultivars are recalcitrant to plant transformation (Young and Udvardi, 2009; Song et al., 2013). Nevertheless, based on existing studies, the possible mechanisms can be proposed to explain the legume reproductive tolerance to heat stress at both physiological and molecular levels (Figure 1).

**Table 2:** QTLs for traits associated with heat tolerance response in legumes.

Crop	Population	No of lines	Marker	Chromosome	Markers	QTL	Traits	Reference
Cowpea	CB27 × IT82E-18, RIL	166	1,536 SNPs	2; 7; 6; 10; 3		Cht-1; Cht-2; Cht-3; Cht-4; Cht-5	The number of pods per peduncle	Lucas et al., 2013
	IT93K-503-1 × CB46, RIL	113	1,536 SNPs	linkage group 8; linkage group 3	1_0032-1_1128; 1_0794_0871	Hbs-1; Hbs-2	Heat-induced browning (Hbs) of seed coats	Pottorff et al., 2014
	IT84S-2,246 × TVu14676, RIL	136		linkage group 5; linkage group 3	1_0032; 1_0280-1_1404	Hbs-1; Hbs-3		
Chickpea	ICC 4567 × ICC 15614, RIL	292	271 SNPs	CaLG05	Ca5_44667768-Ca5_46955940	qfpod02_5; qts02_5; qgy02_5; q%podset06_5	Filled pods; Seed number; Grain yield; %Pod Set	Pronob et al., 2018
				CaLG06	Ca6_7846335-Ca6_14353624	qvs05_6; qfpod03_6; qgy03_6; q%podset08_6	Visual score; Filled pods; Grain yield; m %Pod Set	
Soybean	Natural population	36 varieties	49 SSRs	Gm1; Gm15; Gm16	Sat_201; Sat_452; Sat_380		Flowering time under high temperature	Bai et al., 2015

*Visual score defined in Pronob et al., 2018*.

### Heat Shock Proteins (HSPs) and Heat Tolerance

It is well-known that HSPs play a vital role in plant heat tolerance by maintaining the function of proteins and integrity of various biomembranes under heat stress (Queitsch et al., 2000; Giorno et al., 2010; Fragkostefanakis et al., 2016; Ohama et al., 2016). HSPs act as molecular chaperones, preventing protein denaturation and aggregation. Existing studies have revealed that HSPs can enhance heat tolerance of legumes. For example, the accumulation of HSPs closely positively correlates with heat tolerance of soybean seedlings (Lin et al., 1984). Recently, genome-wide analysis and expression profiles indicate that the *HSP* gene family is involved in drought and heat stress responses in soybean seedlings (Zhang et al., 2015). Proteomic analysis also shows that heat shock-related proteins seem to be regulating heat tolerance in soybean seedlings (Das et al., 2016). It is noted that the above reports are mainly from studies in legume vegetative organs. The accumulation of HSPs in vegetative organs is not related to the heat tolerance of legume reproductive organs (Hall, 2004). Thus, more evidence is necessary in elucidating the roles of HSPs for heat tolerance of legume reproductive organs.

It is reported that five QTLs identified are related to pod set number per peduncle under heat stress (Lucas et al., 2013; Pottorff et al., 2014). Further syntenic analysis with the soybean genome revealed that these QTL regions included HSPs and heat shock factor (HSF) genes. HSFs play an important role in improved tolerance against heat stress by regulating the expression of heat-responsive genes including HSPs (Ohama et al., 2016). Ma et al. (2016) have shown that the *CarHSFB2*, a class B HSF, functions in the developing seed, and the embryo of germinating seed with heat stress responses in chickpea.

### Antioxidants and Heat Tolerance

Heat stress often leads to excess accumulation of reactive oxygen species (ROS) such as superoxide radical (O_2_^−^) and hydrogen peroxide (H_2_O_2_), causing oxidative damage to DNA, proteins, and lipids and thus reproductive failure (Dat et al., 1998; Laloi et al., 2004; Foyer and Noctor, 2005; Oktyabrsky and Smirnova, 2007). For example, heat stress (38/28°C) decreases the activity of superoxide dismutase by 13.3%, catalase by 44.6%, and peroxidase by 42.9% and increases O_2_^−^ by 63.0% and H_2_O_2_ by 70.4% and membrane damage by 54.7% in soybean leaves, compared to optimum temperature (28/18°C) (Djanaguiraman and Prasad, 2010). Increased H_2_O_2_ content and lipid peroxidation under heat stress are also observed in chickpea and mung bean leaves (Kumar et al., 2013; Nahar et al., 2015).

Antioxidants, such as glutathione (GSH), ascorbic acid (AsA) and proline, play important roles in protecting plants from oxidative damage by scavenging ROS and thus enhance heat tolerance of legumes. For example, the application of exogenous GSH enhanced mung bean seedling tolerance of short-term high temperature stress (42°C) by modulating the antioxidant and glyoxalase systems (Nahar et al., 2015). Application of exogenous proline improved chickpea heat tolerance, which is partially attributed to the enhanced biosynthesis of GSH and AsA in shoots and roots (Kaushal et al., 2011). In addition, heat-tolerant chickpea genotypes have shown higher levels of AsA and reduced GSH in leaves than sensitive genotypes under heat stress (Kumar et al., 2013). However, little is known about effects of heat stress on ROS content and oxidative damage in legume reproductive organs. Chebrolu et al. (2016) have found that a diverse set of antioxidant metabolites, including tocopherols, flavonoids, phenylpropanoids, and ascorbate precursors are enriched in the seed of heat tolerant soybean genotypes, based on global metabolite profiles. There is evidence that HSFs may be activated by ROS signaling. In addition to stimulating the expression of HSPs, HSF can also affect ROS scavenger gene expression (Driedonks et al., 2015).

### Compatible Osmolyte and Heat Tolerance

Under abiotic stresses such as heat stress, drought, and salinity, plants often overproduce different types of compatible organic solutes, among which proline and glycine betaine are important in stress tolerance of plants by acting as osmoprotectants and ROS scavengers (Ashraf and Foolad, 2007). In chickpea, exogenous application of proline and glycine betaine improves the growth of seedlings under heat stress (Kaushal et al., 2011; Kumar et al., 2012). Proline may enhance heat tolerance of chickpea through alleviating the inhibition of heat stress on key enzymes in carbon and oxidative metabolism in seedlings (Kaushal et al., 2011). Proline translocation also appears to play an important role in controlling heat tolerance of reproductive development in cowpea (Mutters et al., 1989). Proline transporter genes have been identified among five heat-tolerant QTLs relevant to cowpea reproduction (Lucas et al., 2013). Therefore, it is speculated that proline and its transportation might regulate the response of legume reproduction to heat stress, which will be further testified by more direct evidence.

### Hormones and Heat Tolerance

Hormones play vital roles in plant reproduction under both normal and heat stress conditions. In general, auxin, gibberellin (GA), and cytokinin (CK) positively regulate plant reproductive tolerance to heat stress (Ozga et al., 2017 and references therein). For example, in common bean, the heat tolerant cultivars which have a smaller loss in pod and seed number under heat stress, have a smaller reduction of indole-3-acetic acid (IAA) content in flowers and young pods (Ofir et al., 1993). Furthermore, foliar application of the auxins 4-chloroindole-3-acetic acid (4-Cl-IAA) at early reproductive stage of pea increased its seed yield under heat stress (Abeysingha, 2015).

Different from auxin, ethylene (ET) may play a negative role in legume reproduction under heat stress. Heat treatment of soybean plants increases ET production rate along with induction of oxidative damage, which triggers flower abscission and decreased pod set percentage (Djanaguiraman and Prasad, 2010; Djanaguiraman et al., 2011). The application of the ethylene perception inhibitor 1-MCP (1-Methylcyclopropene) reduces or postpones reproduction failure by inhibiting ethylene production (Djanaguiraman and Prasad, 2010; Djanaguiraman et al., 2011). The positive role of auxin in legume reproduction under heat stress may be attributed to its inhibition of ET-induced floral abscission since the hastened pedicel abscission with exogenous application of ethephon in soybean is inhibited by treatment of exogenous indole-3-acetic acid under normal conditions (Oberholster et al., 1991). Recent research has further revealed that the effects of heat stress on ET biosynthesis and legume reproduction seems dependent on the development stage of reproductive organs. In this research on pea, heat stress promotes ET biosynthesis in the pre-pollinated ovary and thus increases fruit abscission, whereas heat stress inhibits ET biosynthesis in pollinated ovary and consequently it has no significant effects on ovary development (Savada et al., 2017). It is noteworthy, however, that in non-legume crops, ET plays negative roles only at anthesis and post-anthesis stages under heat stress, but positive roles at the pre-anthesis stage (Firon et al., 2012; Ozga et al., 2017 and references therein). Thus, further research is needed to clarify the differences of findings.

The role of ABA in legume reproduction under heat stress remains to be elucidated. The heat tolerance of chickpea seedlings can be enhanced by exogenous ABA application, which leads to elevated accumulation of osmoprotectants including proline and glycine betaine (Kumar et al., 2012). More recently, it has been found that overexpression of the SQUAMOSA PROMOTER BINDING PROTEIN-LIKE transcription factors SPL1 or SPL12 enhances the thermotolerance in both *Arabidopsis* and tobacco inflorescences (Chao et al., 2017). The inflorescence thermotolerance conferred by SPL1 and SPL2 is associated with PYL (ABA receptor)-mediated ABA signaling. Thus, based on these findings at the legume vegetative stage and non-legume reproductive stage, it could be speculated that ABA plays a positive role in heat tolerance of legume reproduction (Ji et al., 2011). To date, few studies have been conducted on the role of hormones in the heat tolerance of legume reproduction.

### Sugar Starvation Responsible for Heat Sensitivity of Legume Reproduction

A large number of studies in non-legume crops have suggested that the high sensitivity of reproductive development to heat stress is attributed to sugar depletion (Frank et al., 2009; Liu et al., 2013; Ruan, 2014 and references therein), probably due to reduced photosynthesis and/or increased respiration (Prasad et al., 2008; Mittler and Blumwald, 2010). In tomato, for example, heat stress decreased the number of pollen grains per flower, pollen viability and germination in tomato, which was attributed to the reduction of starch and soluble sugar content in the pollen grain (Sato et al., 2002). Compared to the sensitive genotype, pollen grains from the heat-tolerant tomato genotype had an unchanged starch and soluble sugar content under heat stress (Firon et al., 2006). Similarly, heat stress compromised the growth rate of pollen tubes through the style of cotton due to an inadequate supply of sucrose and hexose in the pistil (Snider et al., 2009).

Studies on effects of heat stress on sugar content in legume reproductive organs remain scarce. In chickpea, heat-sensitive cultivars have lower sucrose and hexose content in anther and pollen than heat-tolerant cultivars under heat stress, which is related to lower pollen germination rate and fertilization in the heat-sensitive cultivar (Kaushal et al., 2013). Their further study reveals that the activities of sucrose synthase (SUS) and vacuolar invertase (VIN) in these organs is inhibited to a larger extent in heat-sensitive cultivars, suggesting vital roles of sucrose catabolism in controlling heat tolerance of reproductive development possibly through providing hexoses for various structural and functional requirements.

On one hand, to deal with heat stress, plants have to divert considerable resource to heat shock response to maintain normal structure and function of cells, since the biosynthesis of heat shock proteins (Finka et al., 2012; Mittler et al., 2012), antioxidants (Bolouri-Moghaddam et al., 2010; Xiang et al., 2011), and compatible osmolytes has a high energetic and metabolic cost. This could further deteriorate sugar starvation by competing sugars with reproductive organs (Wahid et al., 2007). On the other hand, sugar limitation by heat stress often induces the accumulation of reactive oxygen species (ROS) and subsequently oxidative damage of plant cells (Couée et al., 2006; Bolouri-Moghaddam et al., 2010). A continuous and sufficient supply of glucose may maintain the activity of mitochondria-associated hexokinase, which provides sufficient ADP for the mitochondrial electron transport chain to produce ATP, and consequently avoid ROS overproduction (Xiang et al., 2011).

Improvement of sugar metabolism may help to reduce sucrose starvation in crop reproductive development and thus enhances seed/fruit set. In transgenic tomato, the elevated activity of cell wall invertase (CWIN), one enzyme hydrolyzing sucrose into glucose and fructose, enhances fruit set percentage under heat stress by facilitating sucrose import into the young ovary (Liu et al., 2016). Therefore, similar studies need to be implemented in legumes, in order to sustain their seed setting under global warming. A schematic diagram for the control of reproductive tolerance to heat stress based on sugar starvation is shown in Figure 1.

### Exploring Mechanisms of High Night Temperature Affecting Legume Reproduction

It is noted that high temperature studies have been mainly focused on day temperature rather than night temperature. The latter also dramatically affects male reproductive development of legumes. For example, a high night temperature of 27°C impaired anthers dehiscence in common bean (Konsens et al., 1991). In addition, high night temperature (30°C) leads to small and shrunken pollen and thus 100% pod abortion in cowpea (Ahmed et al., 1992). In soybean, high night temperature (29°C) reduces pollen viability, pollen germination, and pod setting rate, ultimately resulting in lower yield (Djanaguiraman et al., 2013).

Under heat stress, night temperature is usually lower than day temperature and thus represents a moderate heat stress. It has been suggested that responsive mechanisms of plant to moderate and severe heat stress are very different (Yeh et al., 2012; Hancock et al., 2014). For example, mitochondrial co-chaperone MGE2 is a component of the DnaK/HSP70 complex. MGE2 is required for tolerance of *Arabidopsis* to moderate heat stress (35°C), but not for the tolerance to severe heat stress (44°C, Hu et al., 2012). In addition, plants are in a different physiological status at night time as compared to day time (Djanaguiraman et al., 2013; Rieu et al., 2017). Therefore, it is imperative to explore physiological and molecular mechanisms underlying legume seed setting in responses to high night temperature.

## *Medicago Truncatula* as a Model to Study the Molecular Basis of Legume Reproductive Heat Tolerance

**Figure 2:**
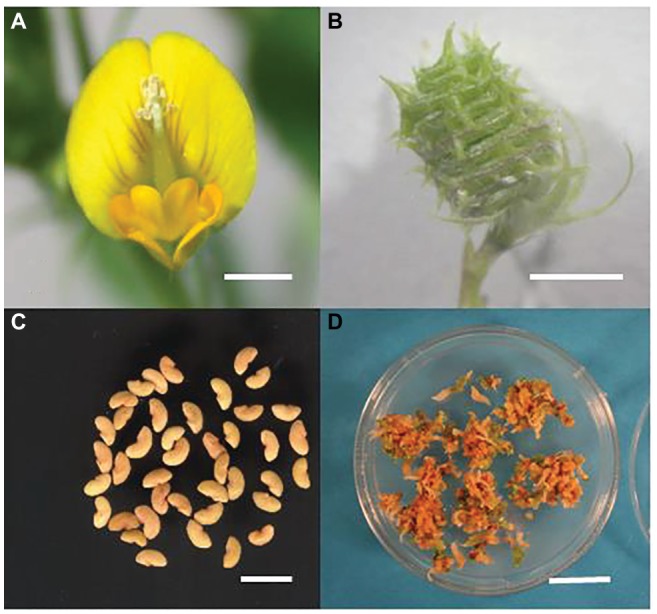
*M. truncatula* flower **(A)**, pod **(B)**, seed **(C)** and highly embryogenic calli used for transformation **(D)**. Bars **(A**,**B,** and **C)** = 5 mm, bar **(D)** = 2 cm. Images from RJR laboratory.

*M. truncatula* has long been used as a model for studying legume biology (Cook, 1999; Rose, 2008). It is further employed to study legume reproductive processes (Figures 2A–C) from development to production (Benlloch et al., 2003; Wang and Grusak, 2005; Chatelain et al., 2012). The anatomy, biochemistry, and molecular profile of *M. truncatula* flower and ovary development have been made clear (Wang et al., 2012; Kurdyukov et al., 2014; Song et al., 2017b). *M. truncatula* has been used to study abiotic stress including heat shock (Soares-Cavalcanti et al., 2012). The late embryogenesis abundant (LEA) proteins have been shown to be related to desiccation tolerance (Boudet et al., 2006). Stress-related genes have been profiled (Shu et al., 2013; Song et al., 2015, 2017; Albornos et al., 2017), and myo-inositol and proline have been shown to be involved in Medicago drought tolerance (Zhang et al., 2014). The salinity tolerance is related to the induction and expression of highly regulated antioxidant mechanisms (Mhadhbi et al., 2011). *MtCBF4* has been shown to play a vital role in abiotic stress responses (Li et al., 2011), and there are 48 candidate *MtERF* genes involved in abiotic stress responses, requiring further functional studies.

*M. truncatula* has a small diploid genome, thoroughly sequenced and annotated (Young et al., 2011; Tang et al., 2014), plus rich available bioinformatic platforms (Benedito et al., 2008; Li et al., 2012a,b; Rose, 2013). It is widely used in functional genomics (Cañas and Beltrán, 2018; De Bruijn, 2019). An efficient transformation system (Figure 2D) has been developed in *M. truncatula* to facilitate gene discovery and functional tests (Song et al., 2013; Nolan et al., 2014). With *M. truncatula* as a model, the mechanisms of heat stress adaptation and tolerance during reproduction may be effectively studied and elucidated in legumes, building on advances in the understanding of epigenetics (Chen et al., 2016), transcriptional regulation (Ohama et al., 2017), and small RNAs (Liu et al., 2017) in relation to heat stress. The findings in *M. truncatula* can be conveniently translated to crop legumes (Young and Udvardi, 2009).

## Conclusions and Future Perspectives

Legume reproductive tolerance to heat stress has been receiving increased attention recently due to legume expansion in adaption to climate change and the demand for sustainable development. This review assessed the reproductive development associated with seed setting in response to heat stress across various legume species. Male development is more sensitive to heat stress compared to female development though both are affected by heat stress. Carbon starvation resulting from reduced photosynthetic supply and carbohydrate diversion for antioxidants, heat shock proteins and osmolytes, is proposed to be a major contributor to reproductive heat response and adaptation. It has been revealed in non-legume crops such as wheat that priming can affect the vegetative stage which helps to improve the thermo-tolerance during the reproductive stage. Priming may have a similar application in legumes, which is deserving of future studies. The clarification of the molecular basis including QTLs of the tolerance of seed setting under heat stress will help accelerate the breeding for reproductive heat tolerance. This can be advanced by the emergence of *M. truncatula* as a model for studying legume reproductive biology, with its well-developed transformation systems and platforms for functional genomics.

## Author Contributions

YL and YS conceived this review, and drafted and finalized the paper. JL, YZ, and AJ helped to improve the draft by providing useful suggestions and information. RR helped in finalizing the paper. All authors approved the work for publication.

### Conflict of Interest Statement

The authors declare that the research was conducted in the absence of any commercial or financial relationships that could be construed as a potential conflict of interest.
